# Mesenchymal Stromal Cells but Not Cardiac Fibroblasts Exert Beneficial Systemic Immunomodulatory Effects in Experimental Myocarditis

**DOI:** 10.1371/journal.pone.0041047

**Published:** 2012-07-17

**Authors:** Konstantinos Savvatis, Sophie van Linthout, Kapka Miteva, Kathleen Pappritz, Dirk Westermann, Joerg C. Schefold, Gerhard Fusch, Alice Weithäuser, Ursula Rauch, Peter-Moritz Becher, Karin Klingel, Jochen Ringe, Andreas Kurtz, Heinz-Peter Schultheiss, Carsten Tschöpe

**Affiliations:** 1 Department of Cardiology and Pneumology, Campus Benjamin Franklin (CBF), Charité – Universitätsmedizin Berlin, Berlin, Germany; 2 Berlin-Brandenburg Center for Regenerative Therapies (BCRT), Charité – Universitätsmedizin Berlin, Berlin, Germany; 3 Department of Nephrology and Intensive Care Medicine, Campus Virchow Clinic (CVK), Charité – Universitätsmedizin Berlin, Berlin, Germany; 4 Department of Pediatrics, University Hospital, Greifswald, Germany; 5 Department of Pediatrics, McMaster University, Hamilton, Canada; 6 Department of Molecular Pathology, University Hospital, Tübingen, Germany; University of São Paulo, Brazil

## Abstract

Systemic application of mesenchymal stromal cells (MSCs) in inflammatory cardiomyopathy exerts cardiobeneficial effects. The mode of action is unclear since a sufficient and long-acting cardiac homing of MSCs is unlikely. We therefore investigated the regulation of the immune response in coxsackievirus B3 (CVB3)-induced acute myocarditis after intravenous application of MSCs. Wildtype mice were infected with CVB3 and treated with either PBS, human MSCs or human cardiac fibroblasts intravenously 1 day after infection. Seven days after infection, MSCs could be detected in the spleen, heart, pancreas, liver, lung and kidney, whereby the highest presence was observed in the lung. MSCs increased significantly the myocardial expression of HGF and decreased the expression of the proinflammatory cytokines TNFα, IL1β and IL6 as well as the severity of myocarditis and ameliorated the left ventricular dysfunction measured by conductance catheter. MSCs upregulated the production of IFNγ in CD4+ and CD8+ cells, the number of IL10-producing regulatory T cells and the apoptosis rate of T cells in the spleen. An increased number of CD4+CD25+FoxP3 could be found in the spleen as well as in the circulation. In contrast, application of human cardiac fibroblasts had no effect on the severity of myocarditis and the systemic immune response observed after MSCs-administration. In conclusion, modulation of the immune response in extracardiac organs is associated with cardiobeneficial effects in experimental inflammatory cardiomyopathy after systemic application of MSCs.

## Introduction

Viral myocarditis is one of the most common causes of inflammatory cardiomyopathy in humans in North America and Europe, especially in children and young adults [1], [2]. It is one of the primary causes of acute heart failure and may progress to dilated cardiomyopathy and chronic heart failure [3]. Many viruses, mainly Coxsackievirus B3 and Parvovirus B19, are thought responsible for a viral myocarditis, which can evolve to chronic myocardial inflammation and inflammatory cardiomyopathy [4], [5], [6], [7].

Till now no specific causal treatment exists for viral myocarditis. Current therapeutic options aim at blocking the renin-angiotensin-system and β-adrenergic receptors, thus slowing down the disease progression and reducing morbidity and mortality [8], [9], [10]. Immunosuppresive interventions might prevent viral clearance and lead to chronic persistence in the myocardium. Therefore an immunomodulatory approach could be able to reduce myocardial inflammation and damage without interfering with the viral clearance.

Mesenchymal stromal cells (MSCs) have been shown to possess immunomodulatory properties. Their immunosuppressive actions on antigen presenting and effector T and B cells have found application in several autoimmune diseases and in organ transplantation, where suppression of the immune system is desirable [11], [12], [13]. In a rat model of autoimmune myocarditis, administration of MSCs attenuated myocardial inflammation through reduced cytokine production and ameliorated cardiac systolic dysfunction [14]. Similarly, we showed in a murine model of CVB3-myocarditis, that intravenous application of MSCs reduced myocardial inflammation and improved cardiac function [15]. However, the involved mechanisms are still not completely understood.

Especially important is the route of administration. Intravenous injection of MSCs exerts cardiobeneficial effects, despite the fact that most of the cells are mainly held in the lung, and some in the liver and organs of the immune system, such as the spleen and lymph nodes [16], [17], [18], [19]. We investigated therefore the hypothesis that extracardiac modulation of immune cells is involved in the cardioprotective effects of intravenous administration of MSCs in CVB3-induced myocarditis.

## Methods

### Animals and Experimental Myocarditis

Male C57Bl/6j mice (6–8 weeks old, n = 60, 10 per group) were purchased from Charles River Laboratories, Sulzfeld, Germany and infected by intraperitoneal injection of 5×10^5^ plaque forming units of CVB3 (Nancy strain) as previously described [15]. Non-infected animals served as controls. The investigation conformed to the Guide for the Care and Use of Laboratory Animals published by the National Institutes of Health and was approved by the local ethics committee (Landesamt für Gesundheit und Soziales, Tierschutz, Berlin).

### Mesenchymal Stromal Cell Isolation and Cell Culture

Human adult MSCs were isolated from iliac crest bone marrow aspirates of normal male donors after their written approval. The aspiration of iliac crest bone marrow was approved by the ethical committee of the Charité-Universitätsmedizin Berlin (EA1/131/07). Aspirates (3–5 ml) were washed twice with phosphate buffered saline (PBS) (Biochrom, Berlin, Germany), and resuspended in low-glucose (1000 mg/l glucose) Dulbecco’s Modified Eagle’s Medium (DMEM; Biochrom) supplemented with 10% fetal bovine serum (FBS), 1% penicillin/streptomycin, 1% glutamine, 2% Hepes and 2 ng/ml of basic fibroblast growth factor (Tebu-bio, Offenbach, Germany). Cells were purified using a percoll gradient at a density of 1.073 g/ml (Biochrom). Next, cells were washed with PBS and then resuspended in complete low-glucose DMEM. Cells were plated at a density of 3×10^5^ cells/cm^2^ and cultured under standard cell culture conditions. Medium was exchanged after 72 hours (h) and every 3 days thereafter. Reaching 90% confluence, cells were trypsinized and replated at a density of 5×10^3^ cells/cm^2^. In order to show that the observed effects are specific for MSCs and are not dependent for the injection of human cells in the mice we used human cardiac fibroblasts as control cells. Human cardiac fibroblasts (Cell Applications, Inc. San Diego, USA) were cultured in Lung/Cardiac Fibroblasts Basal Medium (Cell Applications) plus supplements (Cell Applications). For *in vivo* application, a pool of MSCs from 5 different donors in passage 3 and human cardiac fibroblasts in passage 3 were used.

A number of 10^6^ cells, MSCs or fibroblasts, in a volume of 0.2 ml PBS per animal was administered intravenously through the tail vein on day 1 after infection (n = 10). The same number of cells was administered through the same route and at the same time to a group of uninfected control animals (n = 10). Two further groups of animals, an infected and an uninfected (n = 10 each), received 0.2 ml PBS through the tail vein.

### Flow Cytometry

Flow cytometry analysis of mononuclear cells (MNCs) was performed using directly conjugated monoclonal antibodies (mAbs). All antibodies (CD4, CD8, CD25, IFNγ, IL10), fixation and wash solutions, the Annexin V and the FOXP3 staining kit were purchased from BD Biosciences (Franklin Lakes, NJ, USA). Anti-mouse CD68, CD11c and IFNγ were purchased from BioLegend (San Diego, CA, USA) Surface staining was performed according to the manufacturer’s instructions. Cultured splenocytes were first stained with anti-mouse CD4 and CD25 antibodies, and after fixation, intracellular FOXP3 staining was performed. For intracellular cytokine staining, splenocytes were incubated for 12 h with phorbol myristate acetate (PMA) 50 ng/ml and Ionomycin 500 ng/ml in the presence of BD GolgiStop™ Protein Transport Inhibitor. After the stimulation, splenocytes were first stained with anti-mouse CD4 and CD8, or anti-mouse CD68 and CD11c antibodies, then the cells were fixed in Cytoperm/Cytofix, and stained with anti-mouse IFNγ and IL10 antibodies in BD Perm/Wash solution according to the manufacturer’s instructions. Sample analysis was performed on a MACSQuant Analyzer (Miltenyi Biotec, Bergisch Gladbach, Germany).

### Engraftment Assayed by Real-time PCR

Levels of human MSCs in the myocardium were quantified as described before according to the method of McBride *et al*. [20], slightly modified [21]. In brief, genomic DNA was extracted from frozen tissues as described previously [22]. A standard curve was generated using human genomic DNA obtained from HUVEC serially diluted over a 100,000-fold dilution range, into murine spleen genomic DNA. Real-time PCR was performed with 800 ng of target DNA, Alu specific primers and a fluorescent probe [20]. Values are expressed as % of human DNA per 800 ng of murine tissue.

### Hemodynamic Measurements

Seven days after infection, mice were anesthetized (0.8–1.2 g/kg urethane and 0.05 mg/kg buprenorphine intraperitoneally) and artificially ventilated. A 1.2F microconductance pressure-volume catheter (Scisense Inc., Ontario, Canada) was positioned in the left ventricle (LV) through the apex for continuous registration of LV pressure-volume loops in an open chest model [23]. The volumes were calibrated using the hypertonic saline technique [24]. All measurements were performed three times while ventilation had been turned off momentarily. Indices of systolic and diastolic cardiac performance were derived from LV pressure-volume data obtained at steady state. Systolic function and myocardial contractility were quantified by LV end-systolic pressure, peak rate of rise of LV pressure (dP/dt_max_) and cardiac output (CO). Diastolic performance was measured by the peak rate of LV pressure decrease dP/dt_min_ and the time constant of LV relaxation Tau (τ).

### Tissue Preparation

The heart and spleen were removed and snap frozen in liquid nitrogen and stored in −80°C until further analysis.

### Immunohistology

Immunohistochemistry was carried out on 5 µm-thick cryosections using following antibodies: anti-CD4 (Pharmingen), anti-CD8a (Pharmingen), anti-CD11b (Mac-1) (Pharmingen). Analysis of stained sections was made in a blinded fashion by digital image analysis on a Leica DMRB microscope (Leica Microsystems, Wetzlar, Germay) at a 200× magnification.

### Assessment of Severity of Myocarditis and *in situ* Hybridization

CVB3 positive-strand RNA was visualized in heart tissue using single-stranded 35 S-labeled RNA probes [25]. Pretreatment, hybridization, and washing procedures of dewaxed 5 µm paraffin tissue sections were performed as previously described [26]. Slide preparations were subjected to autoradiography, exposed for 3–4 weeks at 4°C, and counterstained with hematoxylin/eosin. We used a semiquantitative scale with severity scores from 0 to 4 to quantify myocardial damage comprising cardiac cell necrosis, inflammation, and scarring according to Eriksson et al. with minor modification (score: 0, no inflammatory infiltrates; 1, small foci of inflammatory cells between myocytes; 2, foci >100 inflammatory cells; 3, larger foci with an area ≤5% of cross-section involved; 4, >5% of a cross-section involved) [27].

### Real-Time Reverse Transcriptase-PCR

Real-time Reverse Transcriptase (RT)-PCR was carried out as described previously using a 7900 HT Fast Real-Time PCR System, Applied Biosystems, Darmstadt, Germany for the analysis of tumor necrosis factor (TNF)-α, interleukin (IL)-1β, transforming growth factor (TGF)-β, IL10, interferon (IFN)-α, -β, and -γ, and using an Eppendorf Mastercycler epgradient realplex (Hamburg, Germany) for analyzing inducible NO-synthase (iNOS) and hepatocyte-growth factor (HGF) [28]. Primers for TNFα, IL1β, IL6, TGFβ, IL10, IFNs-α, -β, and -γ were bought from Applied Biosystems, whereas primers for iNOS and HGF were custom made. The sequences for murine HGF were forward: 5′-TTGCTCCTCCCTTCCCTACT-3′ and reverse: 5′-TGATCCCTAAAGCAAAGGTCA-3, and for murine iNOS forward: 5′-GGCAGCCTGTGAGACCTTTG-3′ and reverse: 5′-TGCATTGGAAGTGAA GCGTTT-3′. Gene expression of TNFα, IL1β, IL6, TGFβ, IL10, IFN-α, -β, and -γ was measured relative to that of cyclin-dependent kinase inhibitor 1B (CDNK1b) as housekeeping gene. Gene expression of HGF and iNOS was relatively expressed to murine L32 as housekeeping gene. The amount of CVB3-RNA copies in the heart and spleen was quantified by RT-PCR with following primers: forward-primer 5′-CCCTGAATGCGGCTAATCC-3′, reverse-primer 5′-ATTGTCACCATAAG-CAGCCA-3′ (both TIB-Molbiol, Berlin, Germany) and 5-TGCAGCGGAACCG-3 for MGB-TaqMan-probe (Applied Biosystems) as previously described [9].

**Figure 1:**
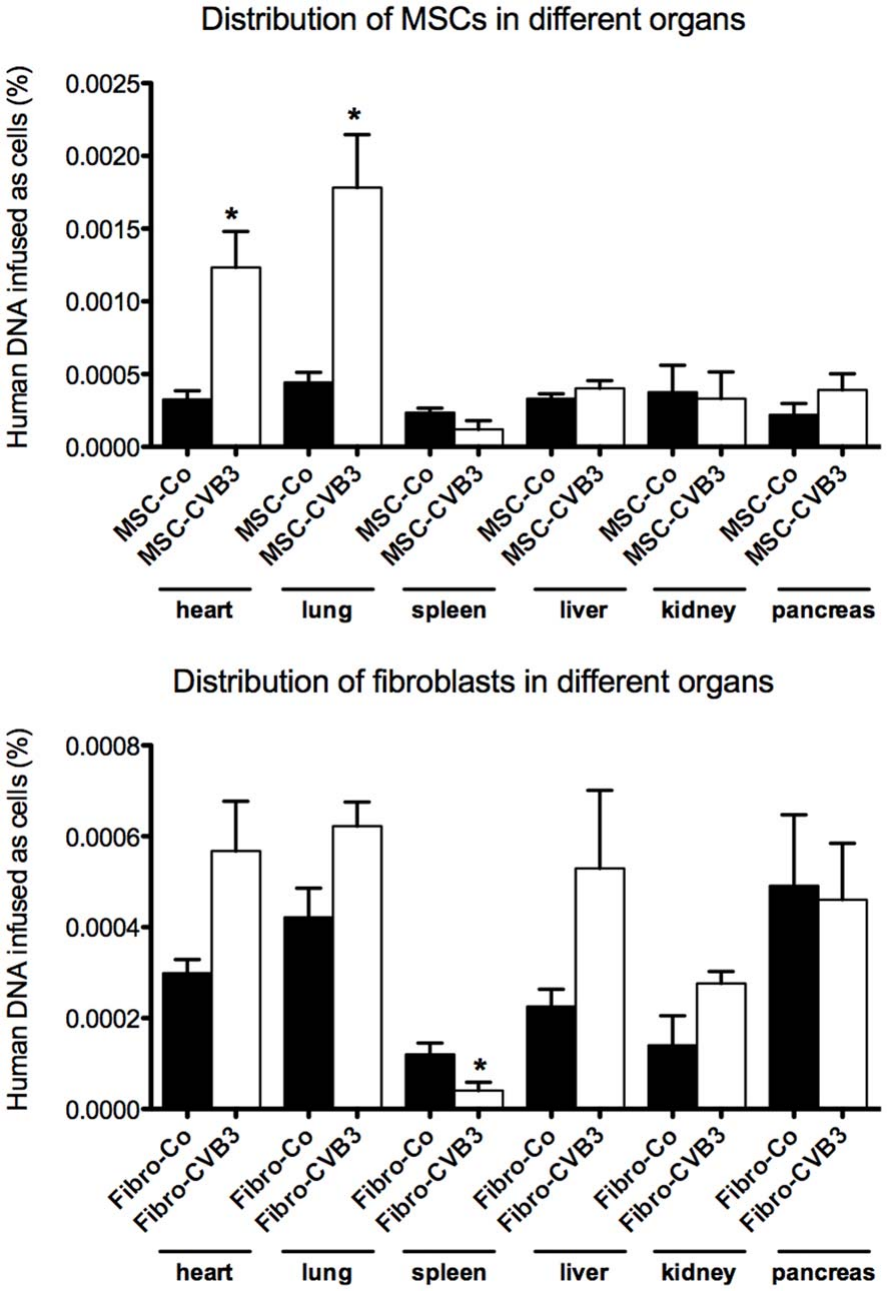
Alu-sequences analysis for localisation of MSCs and cardiac fibroblasts in uninfected and infected mice 6 days after infection. MSCs could be retrieved from the heart, lung, spleen, kidney, liver and pancreas. The highest amount of MSCs was found to be in the lung. Interestingly, the amount of MSCs found in the heart and lung was significantly upregulated after infection. *p<0.05 vs. Control-group.

### Assessment of Myocardial Viral Load with Plaque Assay

To perform plaque assays of CVB3 infected hearts, the tissue was homogenized in 2 ml DMEM followed by three freeze and thaw cycles to destroy the cells and release the virus. HeLa cells were seeded out in 24-well plates and grown to confluence at 37°C overnight. On the next day, cell monolayers were infected with 10fold dilutions of the homogenized heart tissue. After 30 min the solution was aspirated and the HeLa cells were covered with agar containing Eagle’s MEM and FKS as described by Dulbecco [29]. The cells were incubated at 37° for three days and then stained with 0,025% (w/v) neutral red. The virus titer was determined by counting the plaques. Two independent experiments for each heart were performed in duplicates.

### Assessment of Indoleamine Levels and Estimated Indoleamine-Pyrrole 2,3,-Dioxygenase-activity

Serum Samples (100 µl) were measured after addition of 10 µl trichloroacetic acid (50% in water) (FLUKA, Germany), 60 µl deionized water, 100 µl methanol (JT Baker, Deventer, The Netherlands) and 10 µl deuterated standard solutions each (phenylalanine-d_5_, kynurenine-d_6_ and kynurenic acid-d_5_, Cambridge Isotope Laboratories, Andover, MA, USA) was added. The samples were mixed, stored at 4°C over night and centrifuged (20 000 g, 15 min). A Wallac MS2 tandem mass spectrometer (Perkin Elmer, Rodgau, Germany) equipped with an electrospray ion source recorded the levels of trypthophan, 5 times deuterated phenylalanine, kynurenine, 6 times deuterated kynurenine, kynurenic acid, and 5 times deuterated kynurenic acid. Respective Ions were detected in a positive ion mode using multiple reaction monitoring. The two quadrupoles were set to mass-to-charge ratios of (m/z) 205/159 for trypthophan, 171/125 for phenylalanine-d_5_, 209/192 for kynurenine, 215/198 for kynurenine-d_6_, 190/144 for kynurenic acid and 195/149 for kynurenic acid-d_5_. Nitrogen served as collision gas. Flow solvent (0.2% formic acid in 50% aqueous acetonitrile) was set to a flow rate of 50 µl/min. For quantification, plasma samples were spiked with standards. Calibration curves were fitted by linear least-square regression and correlated to the concentration of 5-times deuterized Phe, and 5-times deuterized Kyna as internal standards. Estimated Indoleamine 2,3,-Dioxygenase (IDO)-activity was determined using the Kyn x100/Trp ratio as demonstrated elsewhere [30].

**Figure 2:**
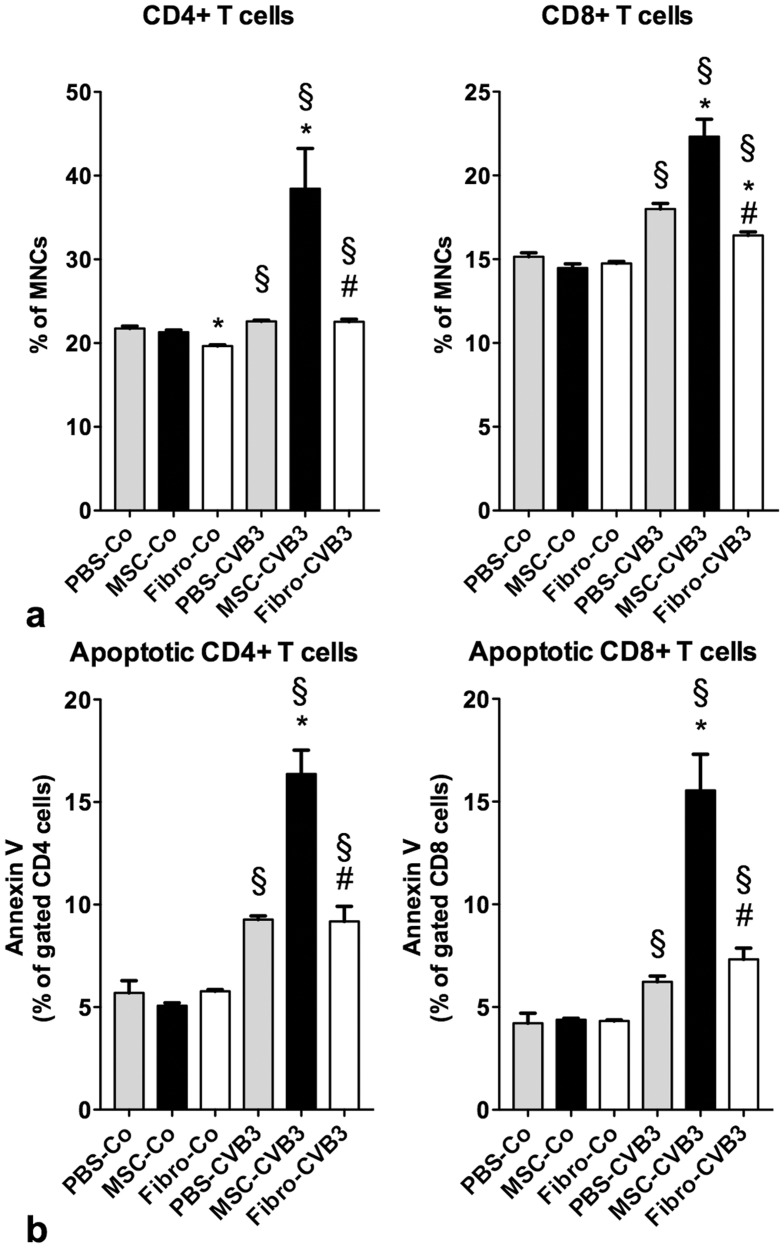
Flow cytometric analysis of splenocytes 7 days after infection. Viral infection led to a significant increase of CD4+ and CD8+ T cells in the spleen, but also induced apoptosis in both cell types. Administration of MSCs led to a significant increase of CD4+ and CD8+ T cells in the spleen in the infected animals compared to the non-treated mice. However, MSCs also significantly increased the apoptosis rate of CD4+ and CD8+ T cells in the infected mice. *p<0.05 vs PBS-CVB3, § p<0.05 vs respective control-group, # p<0.05 vs. MSC-CVB3.

### Splenic IFN-γ and IL-10 ELISA

Splenic IFNγ and IL10 protein levels were determined via the IFNγ (RayBiotech, Norcross, GA, USA) and IL10 (Abcam, Cambridge, MA, USA) ELISA kits, respectively, according to the manufacturer’s protocol. Data are expressed as concentration per µg protein.

**Figure 3:**
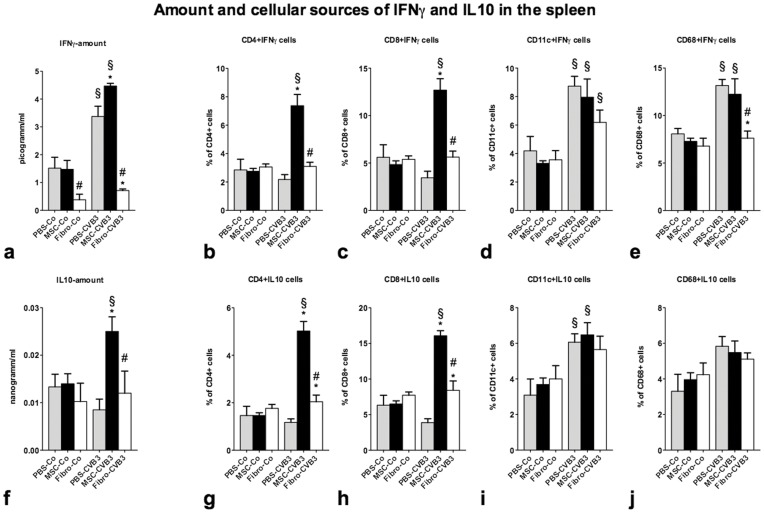
Amount of IFNγ and IL10 measured by ELISA and intracellular staining for IFNγ and IL10 in T cells (CD4+, CD8+), dendritic cells (CD11c+) and macrophages (CD68+) in the spleen 7 days after infection. IL10 and IFNγ were significantly upregulated in the spleen after injection of MSCs. We identified CD4+ and CD8+ T cells as the source of excess IFNγ in the MSC-treated animals (b-c). Furthermore, administration of MSCs led to a significant upregulation of induced regulatory IL10-producing T cells (g-h). *p<0.05 vs PBS-CVB3, § p<0.05 vs respective control-group, # p<0.05 vs. MSC-CVB3.

### Co-culture of Fibroblasts with Splenic Cells

Murine C4 fibroblasts were plated at a cell density of 10,000 cells/96-well in Basal Iscove medium supplemented with 10% FBS and 1% Penicillin/Streptomycin. Twenty-four hours after plating, medium was removed and splenic cells isolated from control+PBS (PBS-Co), control+MSC (MSC-Co), control+cardiac fibroblasts (Fibro-Co), CVB3+PBS (PBS-CVB3), CVB3+MSC (MSC-CVB3), and CVB3+cardiac fibroblasts (Fibro-CVB3) mice were added to the fibroblasts at a ratio of 10 splenic cells to 1 fibroblast in RPMI medium 10% FBS, 1% Penicillin/Streptomycin, and activated with 50 ng/ml PMA and 500 ng/ml ionomycin. After 7 days, the splenic cells were removed and Sirius Red staining was performed.

### Sirius Red Staining

Murine fibroblasts were fixed in methanol overnight at −20°C, washed once with PBS and incubated in 0.1% Direct Red 80 (Sirius red) staining solution at RT for 60 min. After second washing with PBS, the sirius red staining of the human cardiac fibroblast was eluted in 0.1 N sodium hydroxide at RT for 60 min on a rocking platform. The optical density representative for the accumulation of collagen I and III was measured at 540 nm.

**Figure 4:**
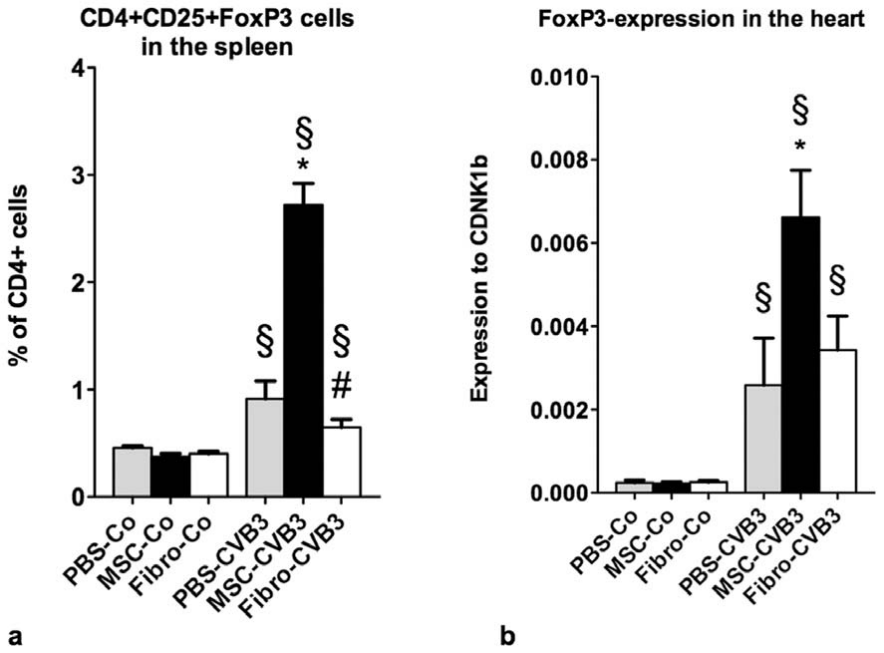
Flow cytometric analysis of naïve CD4+CD25+FoxP3 T cells in the spleen (**a**) **and expression of the FoxP3 transcription factor in the myocardium** (**b**)**.** a. Administration of MSCs induced a significant upregulation of naïve regulatory T cells in the spleen compared to PBS and fibroblasts. b. Expression of the transcription factor FoxP3 in the myocardium was analyzed as an indirect marker for the number of FoxP3-positive regulatory T cells in the heart. Higher expression of FoxP3 in the MSC-treated mice implies a signifcantly higher number of naïve regulatory T cells in the heart compared to PBS- and fibroblast-treated mice. *p<0.05 vs PBS-CVB3, § p<0.05 vs respective control-group, # p<0.05 vs. MSC-CVB3.

### Statistical Analysis

Statistical analysis was performed using Graphpad Prism 5.0 (Graphpad Software, La Jolla, USA). Data are expressed as means ± SEMs. Data were tested for normality with the Kolmogorov–Smirnov and the Shapiro-Wilk tests. Statistical differences betwenn groups were assessed with one way ANOVA and the Bonferroni post-hoc test for normally distributed data or the Kruskal-Wallis test with the Dunn’s post-hoc test for non-parametric data. Differences were considered to be statistically significant at a value of *p*<0.05.

## Results

### Engraftment of Mesenchymal Stromal Cells After Intravenous Injection

We first evaluated the biodistribution of injected MSCs or fibroblasts in the heart, lung, kidney, liver and spleen after intravenous injection via detection of human *Alu* sequences. MSCs could be detected in the heart, lung, spleen, liver, kidney and pancreas 6 days after injection, The injected fibroblasts could also be retrieved in the same organs. Engraftment of MSCs was 4.3 times higher in the heart of infected mice compared to control mice (p<0.05). The highest amount of MSCs could be detected in the lung (Figure 1).

### Effects of Mesenchymal Stromal Cells on the Number and Apoptosis Rate of Splenic CD4+ and CD8+ cells

In order to study the effects of MSCs on the immune system during acute CVB3-myocarditis, we examined spleens from infected and control animals. A suspension of splenocytes was prepared and analyzed with flow cytometry. Administration of MSCs or fibroblasts had no effect on the number of CD4+ or CD8+ T cells in the spleen in uninfected animals (Figure 2a). Infection with CVB3 induced a slight increase of CD4+ T cells (23±0.15% of MNCs, p = 0.04 vs. PBS-Co) in the spleen of untreated mice, which was however further potentiated by MSCs (38±4.8% of MNCs, p = 0.016 vs. PBS-CVB3). The same effects were observed in the CD8+ T cells under infection (18±0.33 vs. 22±1.0, p = 0.007). Administration of cardiac fibroblasts had no effect on the number of CD4+ or CD8+ T cells in the spleen in the infected animals (Figure 2a).

MSCs are known to affect the apoptosis of immune cells [31]. Therefore, we examined the number of apoptotic (Annexin V+) CD4+ and CD8+ T cells in the spleen. Under baseline, we observed no difference in the apoptosis rate of CD4+ (5.7±0.6% in PBS-Co vs. 5.1±0.14% in MSC-Co vs. 5.7±0.08 in Fibro-Co, p = 0.5) or CD8+ T cells (4.2±0.5% in PBS-Co vs. 4.4±0.07% in MSC-Co vs. 4.3±0.05 of CD8+ cells in Fibro-Co, p = 0.9). After infection, apoptosis of CD4+ and CD8+ T cells was increased 1.6-fold (p<0.01 vs. PBS-Co) and 1.5-fold (p = 0.01 vs. PBS-Co) in the PBS-treated mice, respectively. Administration of MSCs caused a marked 3.3-fold increase in the apoptosis rate of the CD4+ T cells (p<0.01 vs. PBS-CVB3) and a 3.5-fold increase of the apoptotic CD8+ T cells (p<0.01 vs. PBS-CVB3, Figure 2b). Apoptosis of CD4+ and CD8+ T cells after infection was not affected by the administration of fibroblasts.

### Mesenchymal Stromal Cells Increase the Production of IFNγ in the Spleen

We and others have previously shown that MSCs require IFNγ to exert their immunomodulatory properties [15]. We therefore analyzed the amount of IFNγ in the spleen and found that administration of MSCs led to a 1.3-fold increase in its production after infection (p = 0.027 vs. PBS-CVB3) (Figure 3a). Interestingly, administration of cardiac fibroblasts led to a significant reduction in the amount of IFNγ in the spleen both in the uninfected and infected animals compared to the PBS-treated mice.

Furthermore, we examined the cellular sources of IFNγ in the spleen. We found that the main sources of IFNγ under infection were CD11c+ and CD68+ cells (Figure 3b–e). However, administration of MSCs led to a significant increase of IFNγ-producing CD4+ and CD8+ T cells (3.4-fold increase of CD4+IFNγ, p = 0.0003 and 3.7-fold increase of CD8+IFNγ T cells, p = 0.0002 vs PBS-CVB3). Administration of cardiac fibroblasts did not affect the IFNγ-production by CD4+ or CD8+ T cells (Figure 3b–e).

### Administration of Mesenchymal Stromal Cells Upregulates the Regulatory T Cell-system

Previous studies have shown that at least some of the effects of MSCs are mediated through upregulation of regulatory T cells [32]. We therefore analyzed the number of naive CD4+CD25+FoxP3 T cells in the spleen. Administration of MSCs led to 3-fold increase of CD4+CD25+FoxP3 T cells in the infected animals (2.7±0.2 vs. 0.92±0.17% of CD4+ cells, p<0.01), while the administration of cardiac fibroblasts had no effect on the number of naive regulatory T cells (Figure 4a).

Another known mechanism of the immunomodulatory actions of MSCs is the upregulation of IL10 and IL10-producing induced regulatory T cells [33]. Analysis with ELISA of the amount of IL10 in the spleen showed a 3-fold increased production of IL10 after MSCs-administration in the infected animals (p<0.01 vs. PBS-CVB3, figure 3f). Furthermore, flow cytometric analysis of the IL10-producing cells revealed CD4+ and CD8+ T cells as sources of IL10 in the spleen. MSCs induced a 4-fold increase in CD4+IL10 T cells (p<0.001 vs. PBS-CVB3) and a 4-fold increase of CD8+IL10 T cells (p<0.001 vs. PBS-CVB3) in the spleen (Figure 3g–j). Administration of cardiac fibroblasts on the other hand led to a slight but significant increase in the number of CD4+IL10 and CD8+IL10 T cells compared to PBS-CVB3. However, the concentration of IL10 in the spleen remained at the same level to that of PBS-CVB3 (Figure 3g–j).

**Figure 5:**
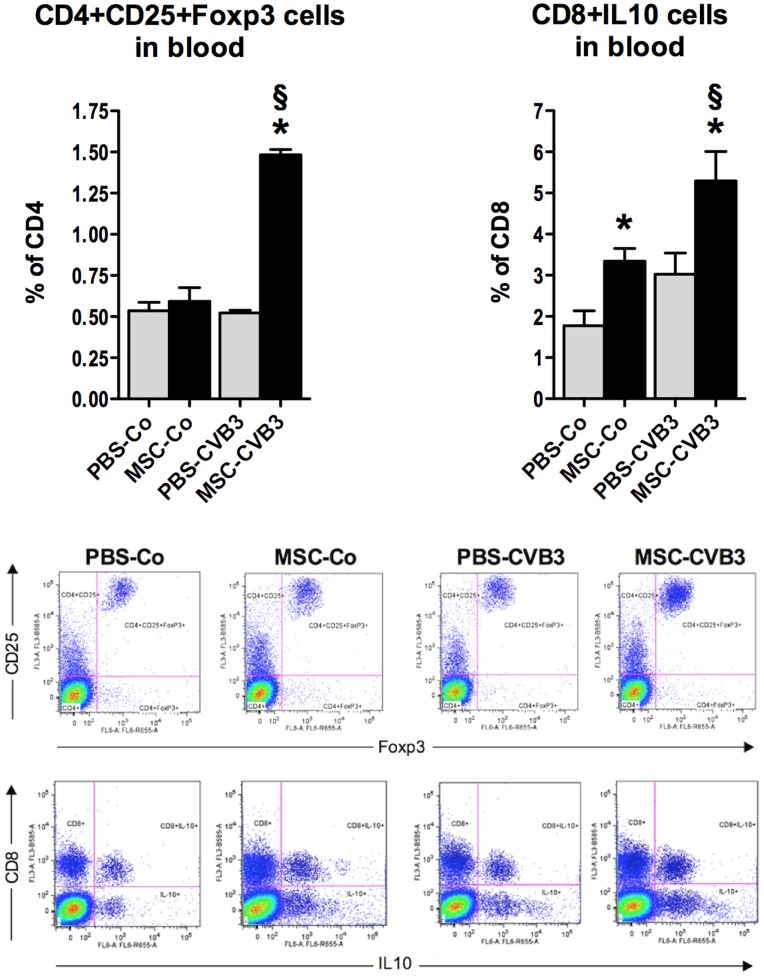
CD4+CD25+FoxP3 and CD8+IL10 T cells in the blood. Similarly to the spleen, naïve and induced regulatory T cells were upregulated in the systemic circulation as an effect of the intravenous injection of MSCs. *p<0.05 vs PBS-CVB3, § p<0.05 vs respective control-group.

We next examined whether the effects of MSCs in the spleen could be observed in the blood as well. The number of CD4+CD25+FoxP3 T cells in the blood was 3-fold increased in the MSC-CVB3 compared to the PBS-CVB3 (p = 0.028). The number of CD8+IL10 T cells in the blood was accordingly increased 1.8-times in the MSC-CVB3 mice (p = 0.043 vs. PBS-CVB3, figure 5).

### Mesenchymal Stromal Cells Attenuate the Severity of Myocarditis

**Figure 6:**
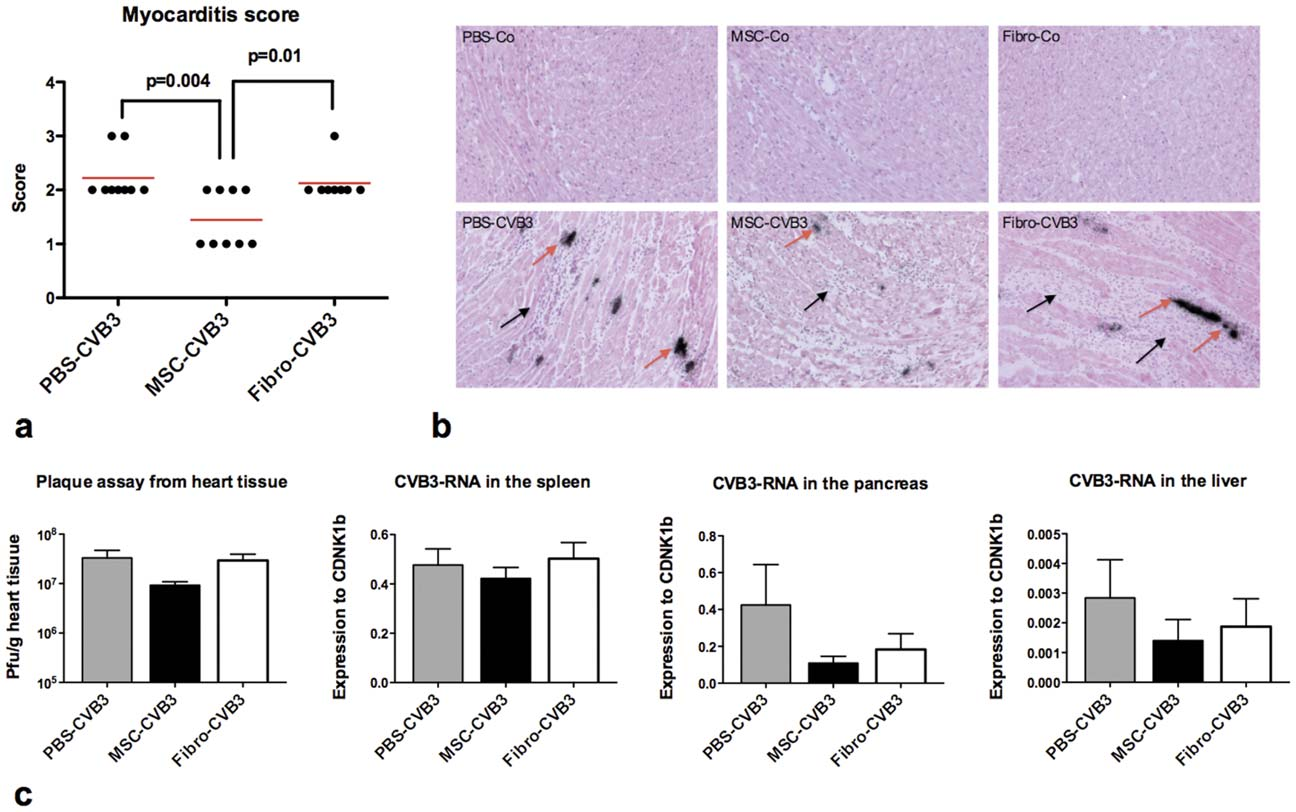
Histopathologic analysis of myocarditis (a-b) and viral replication in the heart (c). a. Pathologic score of the severity of myocarditis assessed by hematoxylin-eosin staining and *in situ* hybridisation of CVB3 positive strand-RNA seven days after infection. b. Representative histological sections of extensive myocardial infiltrations (black arrows) in PBS- and fibroblasts-treated animals and detection of viral RNA in the myocardium (red arrows) (x100 magnification). c. Viral replication assessed by the number of plaque forming units from homogenized cardiac tissue on HeLa cells and viral load assessed by real-time PCR in the spleen, pancreas and liver. MSCs tended to lower not significantly the viral replication in the heart and pancreas compared to PBS and fibroblasts.

Histological sections were stained with hematoxylin/eosin to estimate the extent of myocardial damage and *in situ* hybridisation was used to visualise CVB3 replication in the myocardium. Myocardial cell infiltration, edema and tissue granulation were markedly increased in the infected animals during acute myocarditis compared to the healthy animals. Administration of MSCs reduced the size of inflammatory foci. The pathological score of myocardial damage was significantly higher in the PBS-treated animals compared to the MSCs-treated mice (Figure 6a–b). Severity of myocarditis was not affected by the administration of fibroblasts.

### Myocardial Inflammation is Reduced by Administration of Mesenchymal Stromal Cells

**Figure 7:**
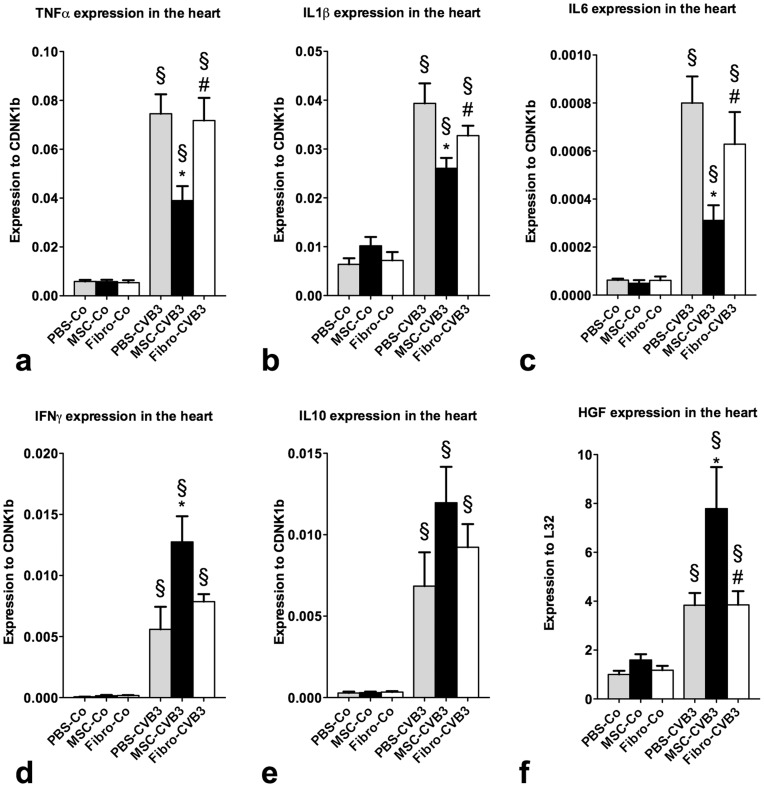
Gene expression of cytokines in the myocardium seven days after infection: Pro-inflammatory cytokines such as TNFα, IL6 and IL1β were significantly upregulated after infection. Administration of MSCs led to a signicant reduction in their expression compared to PBS or fibroblasts. IFNγ, similarly to spleen, was significantly upregulated in the myocardium after treatment with MSCs (d), while IL10 was not affected either by MSCs or fibroblasts (e), Expression of the HGF in the myocardium, a well described mediator of the actions of MSCs was signifantly higher in the MSC-treated compared to the PBS- or fibroblast-treated mice (f). *p<0.05 vs PBS-CVB3, § p<0.05 vs respective control-group, # p<0.05 vs. MSC-CVB3.

In order to investigate the severity of myocardial inflammation during myocarditis, we examined the expression levels of cytokines in the heart. Expression of TNFα, IL1β, IL6 and IFNγ in the myocardium was significantly upregulated in the infected PBS-treated mice compared to the uninfected animals. Administration of MSCs led to a 50% reduction in the expression of TNFα (p = 0.01), a 34% reduction of IL1β (p = 0.02) and a 60%reduction of IL6 (p = 0.006) 7 days after infection (Figure 7a–c). Expression levels of IFN-α and -β, while upregulated after viral infection, did not differ significantly among the infected groups (not shown). Similarly to the observed effects in the spleen, IFNγ expression was 2.3-fold increased in the heart of the MSC-treated infected mice on the 7th day after infection compared to the PBS-treated infected mice (p = 0.03) (Figure 7d). The expression of IL10 in the myocardium remained unchanged after administration of MSCs. Cardiac fibroblasts showed no effect on the expression of the cytokines in the myocardium compared to the PBS-treated infected mice.

### Mesenchymal Stromal Cells Lead to Increased CD4+ and CD8+ T cells in the Heart

We next examined immunohistochemically the infiltration of immune cells in the myocardium. There was no difference in myocardial infiltration of CD11b+, CD4+, or CD8+ cells in the two uninfected groups. On the other hand, there was a marked increase of these cell types in the myocardium of the infected compared to the uninfected mice.

**Figure 8:**
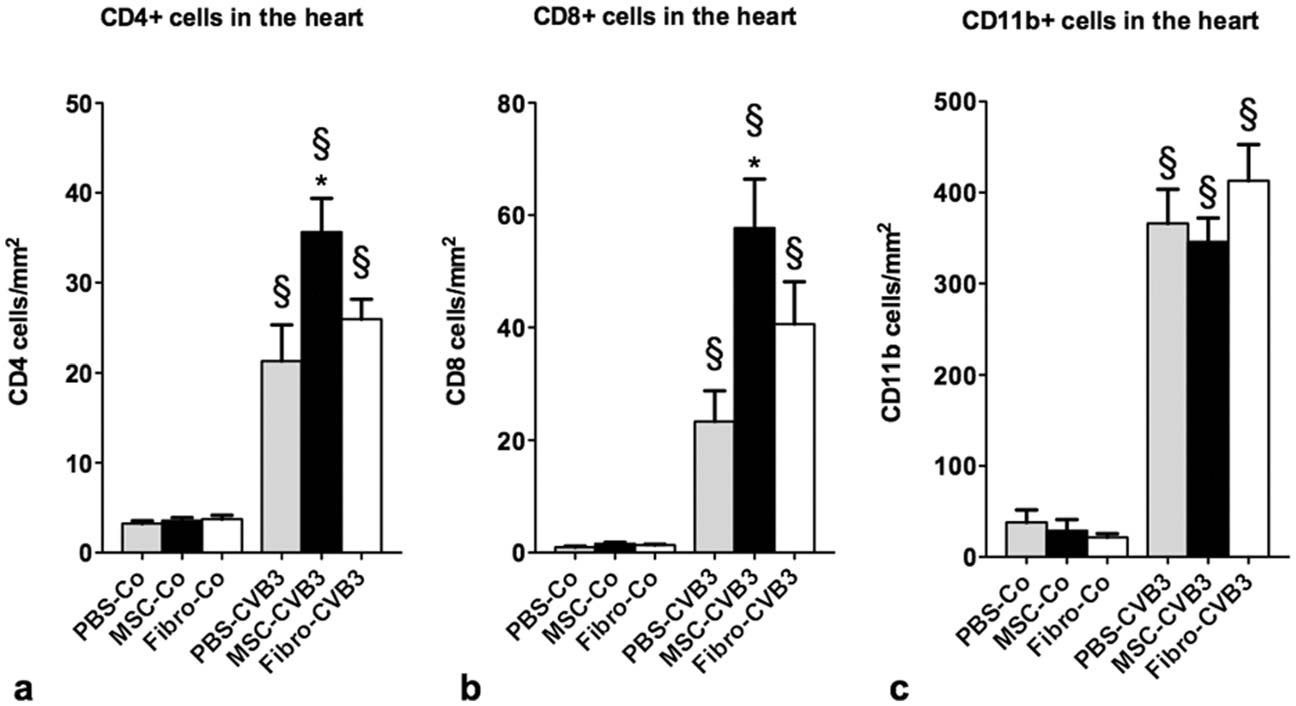
Immune cell infiltration in the myocardium 7 days after infection: a. Macrophages (CD11b+), b. CD4+ T cells and c. CD8+ T cells. *p<0.05 vs PBS-CVB3, § p<0.05 vs respective control-group.

We could not observe any difference in the number of infiltrating CD11b+ cells between the infected groups. However, the number of infiltrating CD4+ and CD8+ cells in the MSC-CVB3 mice was 1.7-times (p = 0.018) and 2.5-times (p<0.01) higher compared to the PBS-CVB3 mice, respectively (Figure 8). The number of infiltrating cells in the myocardium was comparable between the PBS-CVB3 and Fibro-CVB3.

The expression of FoxP3 as an indirect marker for the presence of naive regulatory T cells in the heart revealed a significant upregulation after infection. MSCs potentiated this increase by 2.6 times (p = 0.03), while fibroblasts showed no effect (Figure 4b).

### Mesenchymal Stromal Cells Increase the Cardiac Expression Of Hepatocyte Growth Factor

Several factors have been implicated in the immunomodulatory functions of MSCs, especially TGFβ, HGF, IDO and iNOS [11], [34], [35].

The expression of TGFβ was significantly upregulated in the infected animals compared to the uninfected control mice. However, neither MSCs, nor cardiac fibroblasts showed any effect on its expression in the myocardium. Furthermore, there was no difference in the IDO-activity and iNOS-expression between the infected groups. However, the expression of HGF, was upregulated in the myocardium after infection and we observed a 2-fold increase after injection of MSCs (p = 0.036 vs. PBS-CVB3) but not after administration of cardiac fibroblasts (Figure 7f).

### Administration of human mesenchymal stromal cells ameliorates cardiac dysfunction

In order to examine the effects of MSCs on the cardiac dysfunction during acute myocarditis, we used a microconductance catheter to measure parameters of the LV function on day 7 after infection.

Administration of MSCs 24 hours post infection led to significantly attenuated cardiac dysfunction. Maximal LV-pressure (LVPmax) was reduced by 45% in the PBS-CVB3 after infection (p<0.0001), while administration of MSC led to a 64% higher LVP (p<0.0001 vs. PBS-CVB3). The infected animals showed a 60% reduction in dP/dt max, a marker of cardiac contractility (p<0.0001), while dP/dt max in the MSC-CVB3 was reduced only by 25% (p = 0.0015 vs. PBS-CVB3). The diastolic function was also impaired in the infected mice. The diastolic time constant *τ* was increased by 70% in the PBS-CVB3 mice (p = 0.0002 vs. PBS-Co), while MSCs led to 34% reduction compared to the PBS-CVB3 (p = 0.02 vs. PBS-CVB3). The rate of LV-pressure decrease dP/dt min was similarly increased by 2.4 times in the MSC-CVB3 compared to the PBS-CVB3 mice (p<0.0001).

Finally, MSCs led to a 2-fold increase in cardiac output compared to the PBS-treated infected animals (p = 0.0002).

**Figure 9:**
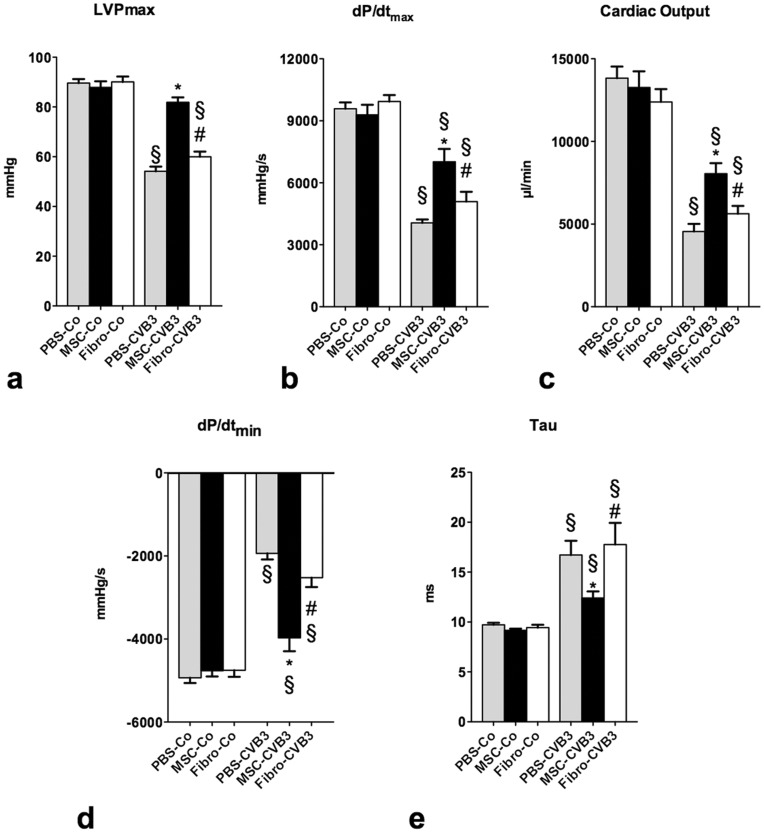
Left ventricular function 7 days post infection, measured by conductance catheter advanced into the heart through thea apex. Administration of MSCs led to a significant improvement of parameters of systolic and diastolic function. As a result cardiac output was also significantly improved. LVP, left ventricular pressure; dP/dt_max_, derivative of left ventricular pressure rise; dP/dt_min_, derivative of left ventricular pressure reduction. *p<0.05 vs PBS-CVB3, § p<0.05 vs respective control-group, # p<0.05 vs. MSC-CVB3.

Administration of cardiac fibroblasts had no significant effect on the cardiac function in the infected animals compared to the PBS-CVB3 (Figure 9a–f).

### MSCs Reduce the Activity of Fibroblasts and the Collagen Content of the Heart

**Figure 10:**
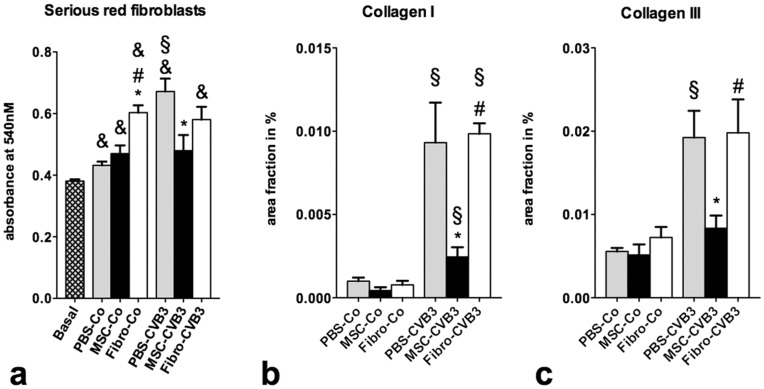
Effects of MSCs on fibroblasts and collagen production during myocarditis. a. Serious red analysis of collagen production by murine fibroblasts co-cultured with splenocytes from infected and healthy mice treated with PBS, MSCs or cardiac fibroblasts. Splenocytes from infected animals induced an increased production of collagen in the fibroblasts compared to those from non-infected mice. However, splenocytes from MSC-treated mice led to a significantly lower activation of murine fibroblasts as measured by their collagen production. b-c. Immunohistochemical analysis of the collagen I and III amount in the myocardium showed a significantly lower myocardial fibrosis after MSC-treatment compared to PBS and fibroblasts and confirmed our in vitro experiments. *p<0.05 vs PBS-treated mice with same infection status, § p<0.05 vs respective control-group, # p<0.05 vs. MSC-treated mice with same infection status, & p<0.05 vs. murine fibroblasts in basal conditions (cultured alone).

Immunohistochemical analysis for collagen I and III fibers revealed a significant upregulation in the PBS-CVB3 mice after infection compared to the uninfected animals. Administration of MSCs reduced the amount of collagen I by 70% (p = 0.04) and collagen III by 55% (p<0.01). Administration of cardiac fibroblasts did not have any effect on the amount of collagen I or III in the myocardium compared to the PBS-CVB3 mice (Figure 10b–c).

Fibroblasts play important role in cardiac remodeling and fibrosis and their function is affected by the interaction with immune cells. Therefore, we examined the effects of splenocytes isolated from infected and healthy mice after treatment with PBS, MSCs and cardiac fibroblasts on collagen production by murine fibroblasts. The addition of uninfected splenocytes significantly increased the collagen production by murine fibroblasts. However, splenocytes from Fibro-Co mice increased the collagen production already under basal conditions compared to PBS-Co and MSC-Co (p<0.0001 vs. PB-Co and SMC-Co).

Splenocytes from PBS-CVB3 mice increased the collagen production by 50% compared to PBS-Co (p = 0.0003). However, splenocytes form MSC-CVB3 mice caused a 30% reduction in collagen production compared to PBS-CVB3 (p = 0.016). Splenocytes from Fibro-CVB3 mice exhibited the same collagen amount to PBS-treated mice (Figure 10a).

### Effects of MSCs on the Inflammation in the Pancreas and Liver

**Figure 11:**
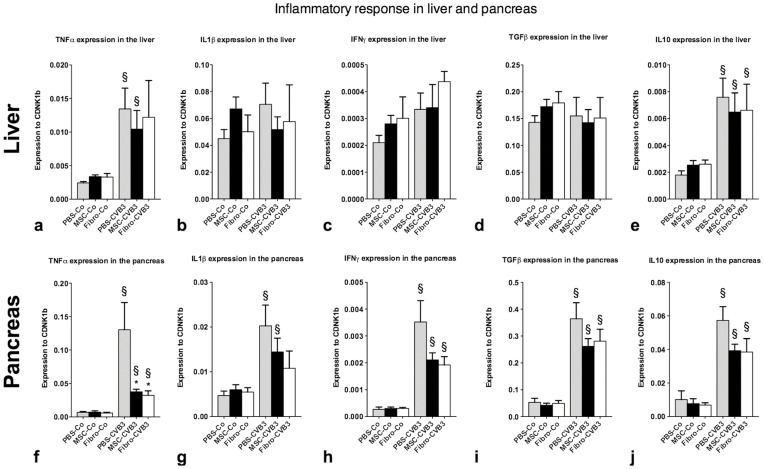
Cytokines expression and analysis of the inflammatory response in the liver and pancreas. The amount of pro- and antiinflammatory cytokines in the liver was the same for all treatment groups. In contrast, expression of TNFα in the pancreas was significantly reduced by both MSCs and cardiac fibroblasts (f). IL1β and IFNγ in the pancreas, tended to be lower after MSCs and fibroblasts-administration but did not reach the significancy level. *p<0.05 vs PBS-CVB3, § p<0.05 vs respective control-group.

CVB3 infection is a systemic disease and affects other organs, such as the liver and pancreas as well [36]. Therefore we examined the effects of MSCs and cardiac fibroblasts by measuring the expression of pro- and anti-inflammatory cytokines in the pancreas and liver 7 days after infection. Furthermore, we analyzed the inflammation score and performed *in situ* hybridization in the pancreas. All infected animals showed a complete infection of the pancreas with a score of 4 and extended necrotic areas. There was no difference between the infected groups. Expression of TNFα, IL1b, IFNγ, TGFβ and IL10 was still significantly upregulated in the infected animals compared to the non-infected 7 days after infection. TNFα expression was 70% lower in both the MSC- and fibroblasts-treated animals after infection (p = 0.02 for MSC-CVB3 and p = 0.045 for Fibro-CVB3 vs. PBS-CVB3). However, no difference was observed in the expression of the other cytokines among the infected groups (Figure 11a–e).

The inflammatory response in the liver showed a similar picture with a significant increase of TNFα and IL10 under infection. However, the expression of IL1β, IFNγ and TGFβ did not differ between the healthy and infected animals. Neither MSCs, nor cardiac fibroblasts showed any effect on the inflammation in the liver.

### Viral Replication in the Heart and Other Organs

In order to examine whether administration of MSCs during CVB3-induced myocarditis had any effects on viral replication in the heart, we examined the formation of plaques on HeLa cells which were cultured with homogenized myocardial tissue from the infected animals. Both MSCs and cardiac fibroblasts had no significant effect on viral replication in the myocardium, while there was a slight tendency for a lower viral replication in MSC-treated mice (Figure 6c).

Furthermore, we analyzed the amount of CVB3-RNA in the pancreas, liver and spleen with real-time PCR 7 days after infection. Since viral load in these organs at this time point has already reached its peak, RT-PCR was chosen for more accurate analysis of CVB3-RNA [37]. We observed no difference in the amount of CVB3-RNA in the spleen, liver or pancreas after administration of MSCs or cardiac fibroblasts. (Figure 6c).

## Discussion

The salient finding of the present study is that systemic immunomodulatory effects are involved in the cardioprotective effects of intravenously applicated MSCs in acute CVB3-induced myocarditis. This involves actions of MSCs in extracardiac immunompetent organs such as the spleen. These effects are unique for MSCs and could not be reproduced by the administration of human cardiac fibroblasts in our study.

As previously shown, administration of MSCs during the acute phase of CVB3-induced myocarditis attenuated the myocardial inflammation [15]. The histological degree of myocarditis and the expression of proinflammatory cytokines were significantly reduced in the animals treated with MSCs. Cardiac dysfunction was accordingly ameliorated after treatment with MSCs as measured by a micro-conductance pressure-volume catheter.

We examined the effects of MSCs on the systemic immune response during the acute phase of myocarditis. The spleen is a large reservoir of immune cells, which are released into the blood stream and get attracted to inflamed tissue, such as the myocardium during myocarditis. Its importance in myocardial inflammation and function is highlighted by the finding that splenectomy or the arrested release of monocytes from the splenic reservoir decreases the recruitment of monocytes into the healing infarct and improves the myocardial outcome [38]. Furthermore, in viral myocarditis, the spleen belongs to the primary infected organs [39]. We therefore analyzed splenocytes using flow cytometry and found that injection of MSCs after infection with CVB3 led to an increased number of CD4+ and CD8+ T cells compared to the untreated mice. However, the apoptosis rate of CD4+ and CD8+ T cells in the spleen was significantly upregulated in the MSC-treated animals. The induction of apoptosis in activated T lymphocytes is a well described effect of MSCs and has consistently been shown in several studies [11], [31]. This proapoptotic action of MSCs towards activated T cells requires an environment of proinflammatory cytokines, such as the spleen during acute CVB3-myocarditis, which potentiates their immunosuppressive actions [40], [41].

Further analysis of splenocytes identified an increased number of CD4+CD25+FoxP3 and IL10-producing regulatory T cells after injection of MSCs as a potential mediator of the immunomodulatory effects of MSCs. Regulatory T cells have immunosupressive functions and are now increasingly recognised as an essential part of the inflammatory response by coordinating the immune reaction and retaining the inflammation leading to its resolution and reducing excessive tissue damage [42]. A study by Shi *et al.* in an experimental model of CVB3-myocarditis demonstrated that the prophylactic transplantation of naturally occuring Tregs could reduce the severity of myocarditis and that these effects were exerted through TGFβ [43]. In our model TGFβ was not significantly affected by MSCs, however we found an increased amount of IL10 in the spleen. FACS-analysis of IL10-producing cells identified CD4+ and CD8+ T cells as the main source of excess IL10 production in the MSC-treated mice. Furthermore, the upregulation of naïve and induced regulatory T types was conferred in the blood as well.

The observed effects of MSCs in the spleen in our model were associated with increased splenic IFNγ production. IFNγ plays an important regulatory role in the actions of MSCs. Priming and activation of MSCs can be facilitated by IFNγ. We and others have shown that IFNγ is necessary for MSCs to exert their immunosuppressive actions [15], [41], [44]. Furthermore, MSCs can upregulate IFNγ production by immune cells, which acts as a positive feedback loop [44], [45]. We could show in our study that IFNγ production could be attributed to CD4+ and CD8+ T cells. Inflammatory environments with low IFNγ levels, such as at the beginning of an infection, can facilitate the immunostimulatory properties of MSCs, such as antigen presentation. However, as the infection evolves, activated T cells produce large amounts of IFNγ, which lead to a change in the actions of MSCs and activation of their antiinflammatory properties [45]. Interestingly, IFNγ was also upregulated in the heart, implying that some of the effects observed in the spleen could be conferred to the heart as well. Moreover a recent study by Koch *et al.* showed, that in a type 1 inflammation environment, IFNγ stimulated the differentiation of a specific subset of Tregs, which was able to migrate to the inflammatory site and exert antiinflammatory effects, optimized for the suppression of Th1 responses [46]. Furthermore, IFNγ produced by Th1 cells is able to stimulate a type I regulatory cells (Tr1) response, characterized by CD4+IL10 producing T cells [47]. The inflammatory response to myocarditis is characterized by a Th1-type immune response, therefore it is possible that the upregulation of IFNγ after MSC-injection was important for the activation of naïve and Tr1 regulatory T cells [36].

An intriguing finding in our study is the increased number of CD4+ and CD8+ T cells in the myocardium despite the lower expression of proinflammatory cytokines. MSCs have previously been shown to lead to the chemotaxis of T cells, in order to suppress their actions [41]. We recently demonstrated that MSC application decreased the proliferation/activation of cardiac mononuclear cells, suggesting that the activity of the retrieved CD4+ and CD8+ T cells in the heart is suppressed [15]. Moreover, like in the spleen, at least some of the CD4+ and CD8+ cells are possibly apoptotic T cells or regulatory T cells, as ahown by the induction of FoxP3-expression in the myocardium after MSC-treatment, as an indirect marker of FoxP3+ regulatory T cells.

In order to understand better the mechanisms that led to an improved cardiac function in MSC-treated mice, we cultured murine fibroblasts with splenocytes isolated from treated and untreated animals. We found that MSC-treated splenocytes were able to reduce the activity of fibroblasts and the collagen production. Furthermore, we performed immunohistochemical analysis of the collagen I and III content of the myocardium after infection. Our findings that MSC-treated infected mice exhibited less fibrosis compared to PBS- or cardiac fibroblast-treated mice confirmed our *in vitro* results. Cardiac fibrosis is an important factor in myocardial remodeling, function and prognosis after an initial damage to the myocardial structure and is merely associated with the action of fibroblasts in the heart [48]. Reduction of myocardial collagen content in our study was associated with an improved LV function.

Finally, we identified HGF as a possible mediator of the immunomodulatory and antifibrotic effects of MSCs in the heart. HGF is a potent growth factor with antifibrotic and antiapoptotic effects. Our findings are in accordance with previous studies, which have shown favorable effects of HGF in different experimental models of cardiovascular diseases, such as myocardial infarction and autoimmune myocarditis [14], [35], [49], [50]. Studies on the actions of MSCs on alloantigen driven mixed lymphocyte reactions showed that MSCs upregulate the expression of HGF after stimulation with IFNγ and TNFα, an environment which resembles the myocarditic environment in the spleen and the heart [51], [52].

Investigation of the cytokines expression and viral infection in extracardiac organs showed no difference of viral infection and inflammation in the liver. Histologic analysis of the pancreas showed severe and equal infection and necrosis in all treated groups. However, there was a significantly higher expression of TNFα in the pancreas of PBS-CVB3 compared to the MSC- and fibroblasts-treated infected mice. RT-PCR analysis of the viral load showed a slightly higher but not significant amount of CVB3-RNA in the pancreas of PBS-treated mice compared to the other groups. Our findings are in accordance with previous studies, which show that cardiac infection and viral replication is controlled by different factors than infection of the liver and pancreas [5].

In our study we used a mouse strain, C57Bl6j, which is not susceptible to chronic myocarditis. However, it is possible that MSCs will be effective in preventing the chronic autoimmune response in susceptible strains as well, since the primary use of MSCs is in chronic autoimmune diseases [53]. There could be some concern regarding an incomplete virus clearance and persistence in the myocardium after treatment with MSCs. However, our present data show that the virus clearance is not inhibited by MSCs. Further studies are needed to investigate the effects of MSCs in susceptible strains of mice to CVB3-myocarditis.

In conclusion, intravenous application of MSCs in acute CVB3-myocarditis leads to important immunomodulatory effects in extracardiac immunocompetent organs, thus affecting myocardial inflammation. Effects on the spleen include the induction of apoptosis in immune cells, which would otherwise invade the myocardium and cause tissue destruction and transfer viral particles into the heart [21], [34]. Furthermore, several studies have shown that the majority of injected MSCs are trapped in the lung and liver, but are still able to exert beneficial paracrine effects [19]. From this point of view intravenous application of MSCs in viral myocarditis and inflammatory cardiomyopathy is an effective intervention to modulate the systemic immune response and thereby improve the cardiac function.
